# Oxidative Stress in Complex Regional Pain Syndrome (CRPS): No Systemically Elevated Levels of Malondialdehyde, F2-Isoprostanes and 8OHdG in a Selected Sample of Patients

**DOI:** 10.3390/ijms14047784

**Published:** 2013-04-10

**Authors:** Sigrid G. L. Fischer, Roberto S. G. M. Perez, Jan Nouta, Wouter W. A. Zuurmond, Peter G. Scheffer

**Affiliations:** 1 Department of Anesthesiology, VU University Medical Center, 6F002, De Boelelaan 1117, 1081HV Amsterdam, The Netherlands; E-Mail: rsgm.perez@vumc.nl (R.S.G.M.P.); 2 EMGO Institute for Health and Care Research (EMGO+), 1081BT Amsterdam, The Netherlands; 3 Knowledge Consortium Trauma Related Neural Dysfunction (TREND), PO box 9600, 2300RC Leiden, The Netherlands; E-Mail: wwa.zuurmond@vumc.nl (W.W.A.Z.); 4 Department of Clinical Chemistry, VU University Medical Center, De Boelelaan 1117, 1081HV Amsterdam, The Netherlands; E-Mails: j.nouta@vumc.nl (J.N.); p.scheffer@vumc.nl (P.G.S.)

**Keywords:** CRPS, oxidative stress, inflammation, MDA, F2-isoprostanes, 8OHdG

## Abstract

Exaggerated inflammation and oxidative stress are involved in the pathogenesis of Complex Regional Pain Syndrome (CRPS). However, studies assessing markers for oxidative stress in CRPS patients are limited. In this study, markers for lipid peroxidation (malondialdehyde and F2-isoprostanes) and DNA damage (8-hydroxy-2-deoxyguanosine) were measured in nine patients (mean age 50.1 *±* 17.1 years) with short term CRPS-1 (median 3 months) and nine age and sex matched healthy volunteers (mean age 49.3 *±* 16.8 years) to assess and compare the level of oxidative stress. No differences were found in plasma between CRPS patients and healthy volunteers for malondialdehyde (5.2 *±* 0.9 μmol/L *vs.* 5.4 *±* 0.5 μmol/L) F2-isoprostanes (83.9 *±* 18.7 pg/mL *vs.* 80.5 *±* 12.3 pg/mL) and 8-hydroxy-2-deoxyguanosine (92.6 *±* 25.5 pmol/L *vs.* 86.9 *±* 19.0 pmol/L). Likewise, in urine, no differences were observed between CRPS patients and healthy volunteers for F2-isoprostanes (117 ng/mmol, IQR 54.5–124.3 *vs.* 85 ng/mmol, IQR 55.5–110) and 8-hydroxy-2-deoxyguanosine (1.4 *±* 0.7 nmol/mmol *vs.* 1.4 *±* 0.5 nmol/mmol). Our data show no elevation of systemic markers of oxidative stress in CRPS patients compared to matched healthy volunteers. Future research should focus on local sampling methods of oxidative stress with adequate patient selection based on CRPS phenotype and lifestyle.

## 1. Introduction

Complex Regional Pain Syndrome (CRPS) is a disabling condition that is usually preceded by a trauma and characterized by excessive pain, sensory disturbances, changes in temperature and color of the affected area and disturbed motor function [1,2]. An exaggerated inflammatory response is suggested to be a key mechanism in the development of CRPS, resulting in oxidative stress and elevated levels of pro-inflammatory mediators [3–6]. It is hypothesized that oxidative stress in CRPS is due to increased exposure to free oxygen radicals and insufficient anti-oxidative defenses [7], and formed the basis for treatment of CRPS with anti-oxidants [8–10]. Free radicals can be generated by various sources, for example exposure to toxins such as cigarette smoke, inflammation as believed in CRPS, but increased levels of free radicals can also result from the physiological aging process [11]. Since direct detection of free radicals is impracticable due to its highly volatile nature [12], several indirect methods have been proposed to examine the levels of oxidative stress *in vivo*. Lipid peroxidation is a mechanism induced by oxidative stress, in which free radicals abstract a hydrogen atom from a methylene carbon in their side chain. Products of this mechanism are, among others, malondialdehyde (MDA) and F2 isoprostanes [11,13]. MDA is the most studied marker for oxidative stress [13], but F2-isoprostanes better reflects oxidative stress because this marker is more specific and is not influenced by dietary intake [14–16]. Furthermore, high levels of free radicals may react with DNA, leading to generation of 8-hydroxy-2′-deoxyguanosine (8OHdG), which has been widely used as a biomarker for oxidative DNA damage. 8OHdG originates from oxidation of the deoxynucleotide pool and is stable and not metabolized in the systemic circulation, and therefore reliable as a marker for oxidative stress [12,17,18]. Assessment in urine is in general preferred because of the short half-life time low stability of the markers during storage and measurements of plasma [11].

Eisenberg *et al.* found elevated levels of MDA and anti-oxidants in serum and saliva of patients with CRPS, therewith establishing a possible involvement of oxidative stress in the disease mechanism of this condition [5]. These findings may provide an explanation for the effectiveness of the free radical scavengers in the treatment of CRPS [9,19]. Dimethylsulfoxide and N-Acetylcysteine are free radical scavengers that are first choice treatment for CRPS in Dutch guidelines, and their therapeutic effect is described in German guidelines for the treatment of CRPS [8,20]. These laboratory findings may be of value for CRPS and stimulate research on therapeutic options based on aberrant inflammation in CRPS.

However, the patients in the study by Eisenberg *et al.* may not be representative for the population of CRPS patients. The studied patient sample consisted of 17 male and 14 female patients, whereas the prevalence of CRPS is highest in females (3–4:1) [21]. Another concern could be the disease duration of included patients, since inflammatory signs and symptoms are most dominant in short term CRPS [22] whereas Eisenberg *et al.* included predominantly patients with longer disease durations. In addition it is not clear, whether or not lifestyle related factors were corrected for in this study (whereby smoking is considered the most important). From population based studies it is known that CRPS patients compared to matched non-CRPS controls are more often smokers and this may bias results. Furthermore the reliability and accuracy of markers for oxidative stress may have influenced the results. Although MDA is a widely marker for oxidative stress, F2 isoprostane is considered to be a more discriminative marker to measure lipid peroxidation [11]. The stability and influence of external factors between various types of specimen differs, whereby markers for oxidative stress are more stable in urine [16]. Saliva on the other hand, is easily influenced by external influences leading to less reliable analysis.

The aim of the present study was to compare levels of highly specific oxidative stress markers, *i.e.*, F2-isoprostanes and 8OHdG in a representative sample of non-smoking female CRPS patients with a short duration of CRPS to age and gender matched healthy volunteers.

## 2. Results

### 2.1. Patient Characteristics

Nine female CRPS patients, mean age 50.1 years (SD 17.0, age range 19–66), median disease duration 3 months (IQR = 1.5; 2–3.5), and nine age and sex matched healthy volunteers (mean age 49.3 years (SD 16.8, age range 23–76) participated in the study. Age differences between patients and matched healthy volunteers ranged from 1 to 10 years (median 3 years). Five patients with an affected upper extremity and four patients with an affected lower extremity were studied. CRPS patients reported pain scores of 5.2 (mean over a week) and 6.2 (during use of the affected extremity). A vivid inflammatory profile with a warmer affected extremity, edema, pain and functio leasa was found in four patients (Table 1). One patient did not provide a urine sample.

### 2.2. Oxidative Stress Markers

No significant difference was found between CRPS patients and healthy volunteers for MDA (mean 5.3 μmol/L *vs.* 5.4 μmol/L; *p* = 0.66), F2-isoprostanes (mean 83.9 pg/mL *vs.* 86.9 pg/mL; *p* = 0.65) and 8OHdG (mean 92.6 pmol/L *vs.* 86.9 pmol/L; *p* = 0.59) in plasma (Table 1).

Also in urine no significant differences were found between CRPS patients and healthy volunteers for F2-isoprostanes (median 117 ng/mmol *vs.* 85 ng/mmol; *p* = 0.61) and 8OHdG (mean 1.4 nmol/mmol *vs.* 1.4 nmol/mmol; *p* = 0.85).

**Figure 1:**
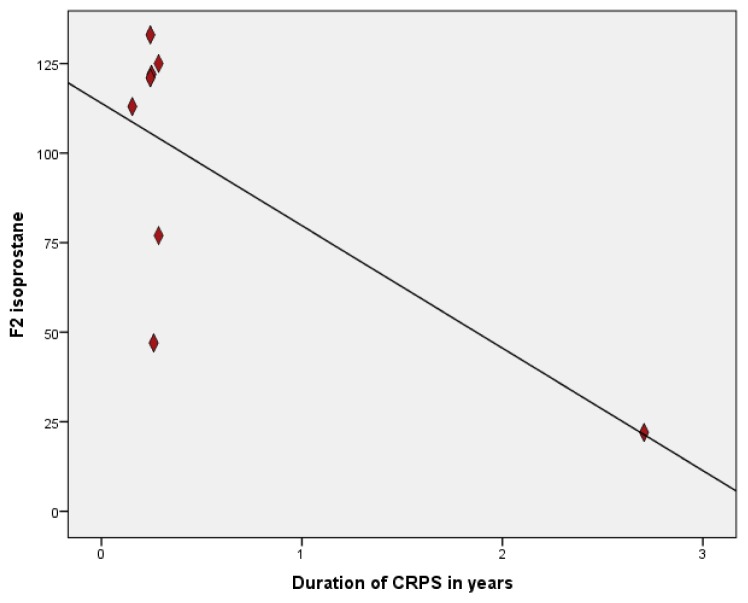
Correlation between levels F2 isoprostane and duration of CRPS. *R* = −0.72; (*p* = 0.04).

Sub-analyses comparing patients with a warm and/or swollen affected extremity to patients with less pronounced inflammatory signs and symptoms showed no significant differences of MDA, F2 isoprostanes and 8 OHdG (*p* > 0.5). However, there was a strong negative correlation (−0.72; *p* = 0.04) between duration of CRPS with levels of F2-isoprostanes in urine, indicating higher levels of oxidative stress in shorter duration of CRPS (Figure 1) Statistically significant correlations between age and marker for oxidative stress were only found for MDA in plasma (0.60; *p* = 0.02), 8OHdG in plasma (0.56; *p* = 0.02) and F2 isoprostanes in urine (0.49; *p* = 0.05). Levels of 8OHdG in plasma and urine were highly correlated (0.80; *p* = 0.00), however for F2-isoprostanes in urine and plasma these correlations were not found (0.29; *p* = 0.26).

## 3. Discussion

In the present pilot study, levels of markers of lipid peroxidation (MDA and F2 isoprostanes) and DNA damage (8OHdG) in plasma and in urine of female CRPS-1 patients were not found to be elevated compared to age and sex matched healthy volunteers. This is in contrast with previous findings whereby elevated levels of MDA were found in serum and saliva [5] in a sample of 31 CRPS patients. This was an unexpected finding since the clinical profile of the included patients in our study was typical for a picture of inflammation (dolor, rubor, calor and functio laesa). Furthermore, a study by Schinkel *et al.* revealed systemically elevated levels of IL-8 and sTNFR in acute CRPS patients [6]. Notwithstanding these findings, the systemic inflammation as expressed by the level of oxidative stress with the markers used in our study did not reveal increased levels of oxidative stress. However, it is too premature to conclude that the well-established theory of tissue injury, inflammation and oxidative stress leading to the development and persistence of CRPS [23–25] should be abandoned. Studies evaluating local cytokines levels and mast cells in blister fluid of the affected extremity reveal elevated levels of TNF-alpha, IL-6 and tryptase as an indicator for increased mast cells in CRPS patients [24–26]. Moreover, diminished oxygen metabolism shown in muscles of affected extremities of 11 CRPS patients [27] and increase of skin lactate as a marker for hypoxia in 11 CRPS patients [28] provided a basis for a localized exaggerated oxidative response in CRPS.

In accordance with our findings, however, is the fact that in several studies evaluating systemic markers no elevated levels of markers for inflammation and oxidative stress were found. Levels of cytokines were found not to be elevated in plasma of nine patients with CRPS, whereas levels of markers of inflammation were increased in blister fluid obtained from the affected CRPS extremity were increased in these same patients [25]. The same result was found in patients with severe CRPS with multiple affected extremities [29].

Another explanation for the disagreement between studies may be the heterogeneity of the population and the selection of a representative patient population. Comparing our present findings to the study by Eisenberg *et al.*[5], several differences already described could have contributed to the differences between both studies. Furthermore, the groups in our study were carefully matched for age in order to minimize differences between the groups (mean 49.3 *vs.* 50.1 years of age, median difference of 3 years), because it is well known that ageing is related to increase of oxidative stress [30,31]. In our studied patient sample, age was strongly correlated to F2-isoprostanes, MDA and 8OHdG. Importantly, in the study by Eisenberg *et al.* more male than female patients were included, no adjustments were performed for smoking and the manner in which age matching was performed was not clearly described.

Additionally, laboratory procedures and specimens differ between studies as well. In the present study both urine and plasma was analyzed, whereby oxidative stress markers in urine are most stable. Moreover a highly specific mass-spectrometry (LC-MS/MS) procedure was used because it has been shown that immuno-assay analyses overestimate levels of F2-isoprostanes and 8 OHdG [28,30]. In the studied samples no correlations were found between levels of F2-isoprostanes in urine and plasma indicating the importance of correct and reliable procedures and difference of stability of markers for oxidative stress. Taken together, the differences in methods and patient populations between both studies may explain the conflicting results that were found.

A limitation of this study is the lack of reference values for products of lipid peroxidation and DNA damage in the general population, leading to the need of matching healthy volunteers, leading to a possible selection bias. The small sample size can also relate to the fact that no significant differences between the groups were found. However, when differences are as small as in our study one should consider its clinical relevance. Notwithstanding these results, discriminative markers for oxidative stress as a screening method for CRPS may be of value for understanding the complex pathology of CRPS and may be helpful in diagnostics and phenotyping of CRPS.

One should keep in mind that measurements have been performed in body fluids representing systemic values of oxidative stress (urine and plasma), while CRPS has a regional distribution [6,25]. Although no support for systemically elevated levels of free radicals was found in this study, one should therefore be cautious in dismissing therapies based on systemic intake of free radical scavengers (*i.e*., N-acetylcystein and vitamin C) as these may exert effects more on a local level. Furthermore, a substantial body of evidence is available for the efficacy of preventing CRPS by increasing vitamin C intake. Nonetheless, considering the fact that both N-acetylcystein and vitamin C may influence multiple biological processes, the efficacy could be related to other mechanisms than attributable to their scavenging properties.

Studies performed to analyze local inflammation and oxidative stress [4,6,25,26] do show positive results in patients with CRPS. It has been shown that most promising markers for research of inflammation in CRPS are pro- and anti-inflammatory cytokines [24,31]. Therefore, another approach should be to assess and compare local inflammatory and oxidative stress markers in individuals and extend research when positive results are found to specify patients with an inflammatory profile of CRPS.

The high correlation found between disease duration and levels of F2-isoprostanes in urine may indicate that patients with a very short disease duration have the most profound inflammatory profile as was suggested in earlier studies [1]. However, these correlations should be considered with care, because of the small sample size potentially biasing our outcomes. Also in our study, one measurement significantly influenced the magnitude of the correlation. Nonetheless, these should be kept in mind when selecting patients for studies evaluating levels of oxidative stress or inflammatory markers in CRPS. On the other hand, studies evaluating anti-inflammatory therapy for patients with CRPS may want to focus on patients with short time CRPS to prevent elaborate tissue injury due to exaggerated inflammation and oxidative stress.

Future research should focus on local assessments of markers of oxidative stress and inflammation, whereby selection of CRPS patients with a short disease duration, an inflammatory phenotype and specific methods of analysis are important. Further research is relevant to elucidate understanding of this complex disease and it may lead to an objective measurement to improve diagnostics, therapy and phenotyping in CRPS.

## 4. Methods

### 4.1. Patients and Healthy Volunteers

Nine female patients with CRPS-1 according to the IASP “Budapest” criteria visiting the pain department of the VU University Medical Center in Amsterdam were enrolled. Blood samples from the unaffected upper extremity and a sample of morning urine were provided. Control samples were obtained from gender and age matched volunteers (<10 years difference). Patients and healthy volunteers did not smoke and did not use free radical scavengers in the week prior to sampling. Blood was collected in EDTA containers and directly centrifuged at 1500 × *g*. Plasma was stored at −80 °C in aliquots. Urine was transferred into polypropylene tubes for storage at −20 °C.

### 4.2. Measurement of Plasma Malondialdehyde

The concentration of total (free and protein-bound) plasma MDA in EDTA-plasma was determined after reaction with thiobarbituric acid (TBA) [32]. To 50 μL of plasma 25 μL of 0.2% butylatedhydroxytoluene (BHT) as anti-oxidant and 0.4 mL 1 mol/L sodium hydroxide for alkaline hydrolysis were added. The mixture was incubated at 60 ° C for 60 min in a shaking water bath. After cooling to room temperature, 1.5 mL of 1% potassium iodide in 10% trichloroacetic acid was added, and the mixture was placed on ice for 10 min and centrifuged at 1500× *g* for 10 min at 4 °C. To 0.5 mL of the supernatant 0.25 mL 41.6 mmol/L TBA was added, and the mixture was heated at 95 °C for 30 min. After cooling to room temperature and centrifugation (1500× *g*, 10 min) 50 μL of the supernatant was injected on a symmetry C-18 column (Waters 4.6 × 100 mm, 3.5 μm) eluted at 1 mL/min by using 70% (*v*/*v*) 25 mmol/L KH_2_PO_4_ (pH 6.8) and 30% (*v*/*v*) methanol. Detection of the MDA-TBA adduct was performed with fluorescence detection (excitation at 515 nm and emission at 553 nm). For quantification the intensities of the MDA-TBA peak areas were compared to standards constructed with tetraethoxypropane (Sigma, St. Louis, MO, USA). The intra-run and inter-run variations were 3.5% and 8.7%, respectively.

### 4.3. Measurement F2-Isoprostanes in Plasma

The total, *i.e.*, free and esterified, concentration of iPF2α-VI was determined by liquid chromatography tandem mass spectrometry (LC-MS/MS). In brief, 0.1 mL of 2 ng/mL deuterated internal standard (8iPF2α-d4; Cayman chemical, Ann Arbor, MI, USA) was added to 0.5 mL EDTA-plasma. Butylated hydroxytoluene (BHT) was added to a final concentration of 0.05% to prevent arachidonic acid from auto-oxidation during sample preparation. Then 0.5 mL of 2.6 mol/L KOH was added, and the samples were incubated for 60 min at 40 °C for alkaline hydrolysis. Afterwards, 250 μL formic acid (20%) was added to adjust the pH at 4.5, and the samples were cleaned up using Oasis mixed-mode anion exchange cartridge (3 cc/60 mg; Waters). The column was successively washed with 2 mL of 2% NH_4_OH, 2 mL 10% methanol 20mM formic acid 40:60, 2 mL 100% hexaan and 2 mL hexaan:ethylacetate 70:30. The fraction containing F2-isoprostanes was eluted with 2 mL of 0.6% acetic acid in ethylacetate and then dried under a stream of nitrogen and successively dissolved in 100 μL 10% acetonitrile containing 0.1% formic acid. A volume of 20 μL was injected on a reverse-phase XTerra MS C18 column (Waters, Milford, MA, USA; 3.5 μm, 2.1 × 100 mm). F2-isoprostanes were quantified by a API 5000 triple quadruple mass spectometer (AB Sciex Technologies, Toronta, Canada). To calculate the iPF2α-VI concentration, the analyte to internal standard peak area ratio with transitions 353.2 and 357.7 respectively to 115.0 were compared with a standard curve up to 8 ng/mL iPF2α-VI (Cayman chemical, Ann Arbor, MI, USA). The intra-run and inter-run variations were 8.1% and 11.3%, respectively.

### 4.4. Measurement F2-Isoprostanes in Urine

The concentration of iPF2α-VI in urine was determined by LC-MS/MS. In brief, 0.1 mL of 10 ng/mL deuterated internal standard (8iPF2α-d4; Cayman chemical, Ann Arbor, MI, USA) was added to 1 mL urine. The sample was then subjected to solid phase extraction (Oasis HLB, Waters, Milford, MA, USA) as previously described [12,33]. The eluate was taken to dryness under a stream of nitrogen at room temperature, and afterwards redissolved in 100 μL 10% acetonitrile of which, 40 μL was injected on a reverse-phase XTerra MS C18 column (Waters, Milford, MA, USA; 3.5 μm, 2.1 × 100 mm). Urinary F2-isoprostanes were quantified using a Quattro Micro (Waters) mass spectrometer. To calculate the iPF2α-VI concentration, the analyte to internal standard peak area ratio was compared with a standard curve from 2 to 16 ng/mL iPF2α-VI (Cayman chemical, Ann Arbor, MI, USA). The intra-run coefficient of variation (CV) was 4.8% and the inter-run CV was 10.1%.

### 4.5. Measurement of 8-Hydroxy-2-Deoxyguanosine in Plasma

Plasma levels 8-OHdG were determined by adding 0.5 mL of 1 nmol/L internal standard (^15^N_5_ 8-OHdG, Buchem, Apeldoorn, The Netherlands) in 3.4% phosphoric acid to 0.5 mL EDTA-plasma. The analytes were then extracted using Oasis mixed-mode anion exchange 96-wells plate (60 mg; Waters, Milford, MA, USA) [34]. Each well was successively washed with 1.5 mL 5% NH_4_OH, 1.5 mL 10% methanol and 1.5 mL 100% methanol. The fraction containing 8OHdG was eluted with 1 mL methanol containing 2% (*v*/*v*) formic acid, dried under a steam of nitrogen at room temperature and redissolved in 100 μL 5% methanol containing 0.1% (*v*/*v*) acidic acid. A volume of 12 μL was injected on a reverse phase HSS T3 column (1.8 μm, 2.1 × 100 mm; Waters, Milford, MA, USA). The eluate components were separated at a flow rate of 0.45 mL/min using a gradient of milliQ water and methanol containing 0.1% acetic acid and were measured on an AB Sciex API 5000 mass spectrometer in positive ion multiple reaction monitoring acquisition mode. To calculate the 8OHdG concentration, the analyte to internal standard peak area ratio with transitions 284.2 to 168.2 and 289.2 to 173.2 respectively were compared with a standard curve ranging 0.2–4.0 nmol/L 8-OHdG. Intra- and inter-assay CVs were 5.8% and 7.2%, respectively.

### 4.6. Measurement of 8-Hydroxy-2-Deoxyguanosine in Urine

Urine levels 8OHdG were determined similarly as determined in plasma, however: urine samples are 10 times diluted in MilliQ water, the standard curve ranges from 1.0 to 16.0 nmol/L [12]. Intra- and inter-assay CVs were 4.1% and 5.3%, respectively.

### 4.7. Statistics

Analyses were performed using SPSS 20. Patient characteristics, levels of F2-isoprostanes, MDA and 8OHdG were compared between patients and healthy volunteers using the independent sample *t* test and Mann-Whitney-U tests. For evenly distributed outcomes values were described as mean and with standard deviation, otherwise outcomes were expressed as median and interquartile ranges (with presentation of 1st and 3rd quartile). Correlations were calculated using Pearson’s correlation between measured markers for oxidative stress and age and disease duration. A *p* < 0.05 was considered significant.

## 5. Conclusions

Altogether, this study does not confirm a role for systemically elevated levels of MDA, F2-isoprostanes and 8OHdG in patients with CRPS in plasma or urine. Although results of this study are based on a small sample size, the selection of patients was representative for the population of CRPS and analytical procedures were highly reliable and specific.

## Figures and Tables

**Table 1:** Characteristic and markers of oxidative stress of Complex Regional Pain Syndrome (CRPS)-1 patients and healthy volunteers.

		CRPS-1 patients	Healthy volunteers	*p*-value
		*n* = 9	*n* = 9	
Age *		50.1 (17.0)	49.3 (16.8)	0.92
Duration CRPS (months) **		3.0 (2.0–3.5)	-	-
Affected extremity		-	-	-
upper/lower		5/4		
Temperature affected extremity (warm/cold/no difference)		4/3/2	-	-
Swelling (yes/no)		4/5	-	-
Reduced range of motion (yes/no)		9/0	-	-

Pain score *	Pain yes/no mean over 1 week during movement	9/0 5.3 (2.3) 6.2 (3.3)	-	-

Impairment levels sum score * (ISS)		26.1 (11.1)	-	-
CRPS Severity Score * (CSS)		10.3 (2.6)	-	-
*Plasma*		-	-	-
MDA* (μmol/L)		5.2 (0.9)	5.4 (0.5)	0.66
F2 isoprostanes * (pg/mL)		83.9 (18.7)	80.5 (12.3)	0.65
8OhdG * (pmol/L)		92.6 (25.5)	86.9 (19.0)	0.60
*Urine*		*n* = 8	*n* = 9	-
F2 isoprostanes ** (ng/mmol)		117 (54.5–124.3)	85.0 (55.5–110.0)	0.61
8OHdG* (nmol/mmol)		1.4 (0.7)	1.4 (0.5)	0.85

* mean and SD (independent sample *t* test).

** median and IQR (Mann Whitney).
